# Individual optimization of risky decisions in duration and distance estimations

**DOI:** 10.3758/s13414-020-02225-6

**Published:** 2020-12-29

**Authors:** Robbert van der Mijn, Atser Damsma, Niels Taatgen, Hedderik van Rijn

**Affiliations:** 1 Department of Experimental Psychology, University of Groningen, Groningen, Netherlands; 2 Behavioral and Cognitive Neuroscience, University of Groningen, Groningen, Netherlands; 3 Department of Artificial Intelligence, University of Groningen, Groningen, Netherlands

**Keywords:** Decision making, Response time models

## Abstract

Many everyday decisions require an accurate perception of how much time has passed since a previous event. Although humans estimate time intervals with a high degree of mean accuracy, the precision of estimations varies greatly between individuals. In situations in which accurate timing is rewarded but responding too early is punished, the optimal amount of risk is directly dependent on the precision of the timer. Previously, it was found that humans and rodents displayed near-optimal adjustment of their mean response time based on their individual precision and the level of punishment. It is as of yet unknown whether these strategies of optimality in interval timing are specific to the timing domain, or instead reflect an ability that generalizes to other sensorimotor modalities of decision making. Here, we address this by combining a temporal reproduction experiment and a distance estimation experiment with an identical reward scheme. We found that participants approached optimality in both tasks, but generally underadjusted their responses in the face of high risk. As this individual adjustment was consistent over modalities, these results can best be explained by assuming that the adjustment of behavior towards optimal performance is driven by a modality independent mechanism.

The last 27 Olympic finals of the 100-meter sprint for men were won with an average difference of 87 milliseconds. Therefore, an athlete waiting for the *go* signal to start a race will aim to respond as fast as possible to get an early advantage. Usually, the *go* signal is given after a variable but predictable amount of time has passed since “*ready . . . set . . . .*” An athlete with an accurate representation of this time interval, and who is able to reproduce it, could take the lead right at the beginning of the race. However, the decision about when to start moving is marked by both external and internal uncertainty. Externally, some uncertainty exists about the duration between “*set . . .*” and “*go*,” which will be somewhat predictable but can vary between races (i.e., due to a different referee, or randomly generated starting signals). Internally, uncertainty arises due to the noisiness of the athlete’s ability to estimate time (Gibbon, Church, & Meck, 1984; Jazayeri & Shadlen, 2010; Maaß & van Rijn, 2018). Given this uncertainty, aiming to move exactly at the start signal may result in a false start. To avoid the risk of disqualification, an optimal athlete might therefore take his or her timing precision into account, and prepare for a slightly later start.

When temporal precision is a significant source of noise, probabilistic reasoning may be used to obtain optimal results. This type of behavior adaptation can be observed in a paradigm in which rewards to a response are only given when a certain duration, referred to as the schedule, has passed since the previous response (Çavdaroğlu, Zeki, & Balcı, 2014; differential reinforcement of low rates [DRL] paradigm). In this paradigm, a too-early response resets the waiting period, resulting in a sudden decrease of reward rate, but the reward rate also decreases with every time unit that the response is later than the schedule. Typical results indicate that participants with noisier response time distributions respond later compared with the more precise timers. This is a signature of rational adaptation, as their noisier response times would more often trigger a costly reset of the waiting period if the aim of their response times were closer to the target duration. Similar behavior can be observed in other tasks (see for a review Freestone & Church, 2016). For example, in a beat-the-clock task, participants are rewarded for responding temporally as close to the schedule as possible, but not later. This task also results in participants aiming for a response before the target duration, with the underestimation determined by a participant’s temporal noisiness (Simen, Balcı, deSouza, Cohen, & Holmes, 2011).

A statistical decision-making theory of time estimation can describe the timing strategy to arrive at an optimal timing plan (Balci et al., 2011; Hudson, Maloney, & Landy, 2008). The *expected gain* (EG) of a temporal aim point *S* is determined by the individual gains (*G*_*i*_) that are associated with the potential time points at which a response could be given (*T*_*i*_) multiplied by the probabilities of responding at those time points given *S* and its standard deviation. In the DRL task described in Çavdaroğlu et al. (2014), the maximum reward rate for a perfect timer (e.g., an ideal but unrealistic timer who can produce durations without any noise) is achieved by responding exactly at the scheduled time. However, the expected gain for aiming at exactly this time point is lower for a more realistic, slightly noisy timer with a symmetrical response distribution: Because of the .5 probability to respond *before* the target duration due to noise, this realistic timer would receive costly punishment in half of the trials. The maximum expected gain is therefore associated with a temporal aim that is shifted towards a later point in time, with the magnitude of the shift depending on the magnitude of the precision. A graphical example of the expected gain function is shown in Fig. 1. In their review, Freestone and Church (2016) emphasized the complexity of the statistical inferences required for optimal timing and discuss that some animals do not account for their own noisiness, whereas humans are reported to earn close to the maximum gain given their precision.

**Fig. 1:**
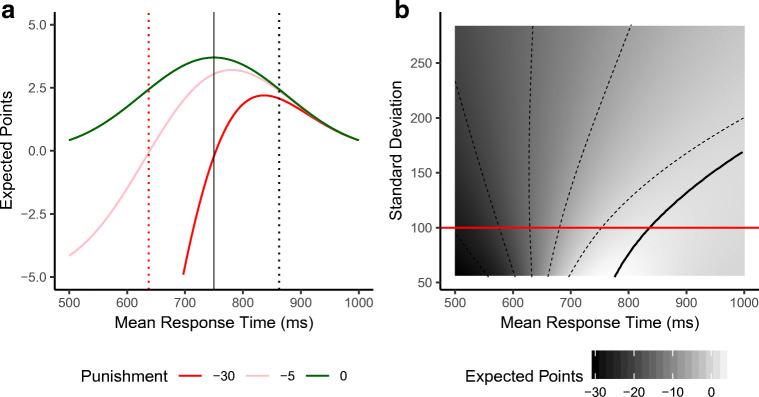
**a** Example of the mean expected points for a participant with a Gaussian response-time-based standard deviation of 100 ms in a task in which reproducing a 750-ms interval within a margin of 30% is rewarded with 5 points, while being too early or late, respectively, results in penalty points of no points. The green line reflects the expected outcome for different mean response times in blocks without penalty: The highest possible reward is achieved when this participant aims for intervals of 750 ms. As the punishment increases to 5 or 30 points, reflected by the pink and red lines, the expected gain peak for this participant decreases and the optimal mean response time shifts towards the right. **b** Surface plot of the expected reward for different means and standard deviations of the Gaussian response-time distributions in a block with 30-point punishment for too-fast response. The red line represents a participant with a standard deviation of 100 ms as depicted in Panel **a**. The black, solid line indicates optimal mean responses as a function of the standard deviation in the −30 block. The optimal response time for the participant with a 100-ms standard deviation is defined by the intersection of the red and black line. (Color figure online)

The integration of risk into decision-making processes has been described extensively (Birnbaum, 2008; Diederich & Trueblood, 2018). Before that, the early model of expected utility (EU) described how decision-makers seek to maximize expected utility: The product of the probability of a certain outcome and the value of that outcome (Bernoulli, 2011/1738). However, this model does not always capture observed behavior. For example, when an outcome is framed as a potential loss, this will lead to a different decision compared with when it is framed as a gain (Tversky & Fox, 1995). This bias was accounted for by prospect theory (Kahneman & Tversky, 1979; Trepel, Fox, & Poldrack, 2005), which explains these differences in terms of risk-seeking or risk-avoiding tendencies. For example, a risk-avoiding participant, deciding between two monetary gambles with the same expected value, might avoid the prospect in which the chance of a negative outcome, irrespective of its value, is higher. It has been proposed that a dynamic combination of two decision systems drive the outcome of these decisions: System 1 is associated with fast and automatic decisions, whereas System 2 is characterized by deliberate and rational decisions (Diederich & Trueblood, 2018; Evans, 2008; Kahneman & Frederick, 2002). Kahneman and Frederick (2002) assume an initial decision is made by the fast System 1, which can be overruled by the rational System 2. This dynamic combination can play a role when decisions are made based on uncertainty that arises from a decisions-makers’ own impreciseness, such as noise in timing judgements.

In everyday tasks, timing is not the only source of internal uncertainty, as any form of external response will be affected by motor noise. Indeed, earlier work has demonstrated that humans take the likelihood of success into account when performing a motor task in which uncertainty is defined by manual motor precision (Maloney, Trommershäuser, & Landy, 2007). In their study, participants were instructed to quickly touch a small reward region on a touch screen to earn points. The deadline for the speeded response was set so low that participants would sometimes miss the target. In addition to a target region, participants were also informed about the existence of a punishment region. Analogous to the expected gain calculations discussed earlier, the location of the punishment region and the magnitude of the punishment determine, together with motor precision, the location of the *maximum expected value*. The behavioral profiles of the participants indicated that they indeed adjusted their movement plans to aim for that location.

Studies that investigate planning optimization, as the examples discussed above, suggest that humans can adjust their responses to maximize their gains in line with Bayesian decision theories (for a review, see Körding & Wolpert, 2006). These types of Bayesian frameworks have been used to understand a variety of magnitude judgements, such as distance, angle, and time estimations (Petzschner, Glasauer, & Stephan, 2015). In the field of timing, for example, it has been shown that participants’ clock accuracy determines the extent to which they weigh prior experiences, with noisier clock measurements associated with a stronger influence of the prior knowledge (Cicchini, Arrighi, Cecchetti, Giusti, & Burr, 2012; Hallez, Damsma, Rhodes, van Rijn, & Droit-Volet, 2019; Maaß, Riemer, Wolbers, & van Rijn, 2019) and affected integration of perception and prior knowledge in clinical populations (Maaß et al., 2019).

Each of the timing and movement preparation studies mentioned above focuses on a single modality (for other modalities, e.g., distance, see Healy, Tack, Schneider, & Barshi, 2015; Petzschner & Glasauer, 2011), with the proposed mechanisms just applying, either implicitly or explicitly, to the specific modality under study. However, all provided explanations assume integration of perceptual and motor noise with potential outcomes. If risk strategies are stable across modalities, the performance of a participant in one domain should predict the participant’s performance in another domain when the differences in domain-specific noise are taken into account. To test this notion, we report on an experiment in which participants performed a timing task and a distance estimation task. In both tasks, participants could earn money by responding accurately, but could, in some experimental blocks, lose money for responding too early (in the timing task) or too close to the reference location (in the distance estimation task). In both tasks, we determined, for each participant, how much adjustment of the mean response from the no-punishment block is required, based on their endogenous noise in the penalty block, to reach the optimal strategy in that penalty block. Thus, optimality is operationalized as the difference between the observed adjustment in the 5 and 30 penalties blocks, and the theoretical adjustment associated with optimal responses. This approach allows us to compare optimality, independent from possible response biases, in the timing task and the distance estimation task.

We hypothesize that participants will respond later/further from the reference in the blocks with punishment. Importantly, we expect the magnitude of this adjustment to be dependent on a combination of their precision and their risk-seeking or risk-avoiding tendencies. We hypothesize that the degree to which a participant is optimal in these adjustments is stable across modalities and therefore correlated. For example, a participant who only adjusts timing response 20 ms, while an optimal shift would have been 40 ms, is expected to underadjust by 50% in the distance estimation task, too.

## Methods

### Participants

Thirty-six participants (age *M* = 24.8 years, *SD* = 4.3 years) volunteered in exchange for €7–10, depending on their performance on the tasks. Participants were recruited from the payed participant pool of the University of Groningen. The study was approved by the Psychology Ethical Committee of the University of Groningen (17129-SP-NE), and participants signed a form giving their informed consent.

### Materials and procedure

Participants performed a timing task and a distance-estimation task, of which the order was counterbalanced between participants. In each task, participants tried to earn as many points as possible (each point was worth 0.69 eurocent). The accumulation of points and money was shown to the participants during the course of the experiment.

#### Timing task

Stimuli were presented on a 12-inch touch-screen monitor (SCHURTER Electronics) using OpenSesame (Mathot, Schreij, & Theeuwes, 2012), and responses were made with the left or right rear buttons of a gamepad (Microsoft Sidewinder), depending on the participants’ preferred hand. Since the viewing distance was not precisely controlled, the descriptions of stimuli contain only estimations of visual angle. All code needed to run this experiment is publicly available (https://osf.io/f68wm/).

**Fig. 2:**
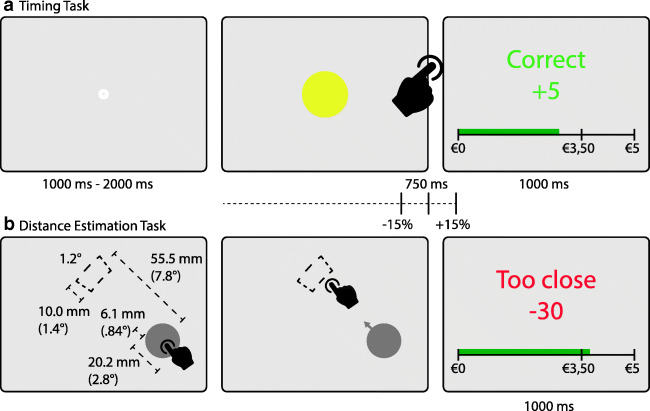
Typical procedure of a timing trial (**a**), in which the participant responded within the 30% margin and received 5 points, and a distance estimation trial (**b**), in which the participant responded too close and 30 points were subtracted. Figure **b** shows an example of a possible configuration of start circle, arrow, and target. The target is represented in dashed lines and was not visible to the participant, except in the instruction screen at the beginning of each block. Between trials, only the angle at which the arrow points and the position of the circle on the screen changed. The green bar in the bottom of the feedback screen indicated the amount of money accumulated over the course of the experiment. (Color figure online)

Participants performed an interval reproduction task, in which they had to reproduce a single interval of 750 ms. First, five passive *learning trials* were presented, in order to become familiar with the target interval. In each trial, participants were first shown a white intertrial fixation circle with a 3-mm diameter (0.46 ° visual angle) for a random duration between 1,000 ms and 2,000 ms. Next, a yellow circle with an 18-mm diameter (visual angle of 2.6° visual angle) was on the screen for 750 ms, then disappeared for random duration between 1,000 ms and 2,000 ms. Subsequently, during the *experimental trials*, participants were asked to reproduce the previously learned interval. The trial procedure was similar to the learning trials; the reproduction interval was initiated by the appearance of the yellow circle, and the participant marked the offset with a gamepad button press. Feedback on whether the produced interval was too long, too short, or within the 30% margins of 750 ms was presented for 1,000 ms. An example of the trial procedure is shown in Fig. 2a.

Equally spread over five experimental blocks, 250 intervals were produced, preceded by a practice block of 50 trials. In the experimental blocks, participants earned 5 points for responding within the 30% margin. In Block 2 and Block 4, points would be deducted if the response was too early. Before each block started, the payoff scheme was presented on the screen. The payoff scheme is shown in Table 1. The total number of points and money collected was shown together with each trial’s feedback.

**Table 1:** Payoff scheme, no points are earned during the practice (P) block

Block	P	1	2	3	4	5
Too early/close	0	0	−5	0	−30	0
Correct	0	5	5	5	5	5
Too late/far	0	0	0	0	0	0

#### Distance estimation task

Participants did a distance estimation task on the same touch-screen monitor as used for the timing task. Each trial started with a “start circle” presented at a random location below the right diagonal for right-handed participants and below the left diagonal for left-handed participants. After touching the start circle, an arrow appeared originating from the start circle, pointing to an invisible target that was always 55 mm (7.9° visual angle) away from the center of the start circle at a random angle (see Fig. 2b). The participant then tried to touch the invisible target to earn points. Feedback was presented on whether the tap was a hit, too close, or too far away relative to the start circle. Also, the accumulation of money was shown during the feedback screen, followed by a 1,000-ms to 2,000-ms intertrial period. In order to learn the distance, participants were shown a visible target once at the typical distance from the start circle during the instructions at the beginning of each block.

Participants performed the distance estimation 250 times, equally spread over five experimental blocks, preceded by 50 practice trials. The procedure regarding points and feedback was identical to the timing task: Participants earned 5 points for touching the target, but points would be deducted in Blocks 2 and 4 if their response was too close to the start circle (see Table 1).

### Analysis

Response times and distance estimations that deviated more than three median absolute deviations from the median, pooled over all trials per participant, were excluded from the analysis (2.7% of the timing trials, 2.1% of the distance estimation trials).

Three hierarchical linear mixed-effects models (LMMs) were performed using the *lme4* package (Version 1.1-14; Bates, Mächler, Bolker, & Walker, 2014) in R (Version 3.6.0; R Core Team, 2018). Separate models for timing and distance estimation were estimated to test the effect of punishment level and the precision of the timer on adjustment of mean response. Adjustment was calculated as the difference between mean responses of the blocks with punishment and Block 1 and were used as dependent continuous variables (in seconds for the timing task and mm for the distance estimation task). Punishment level was added as a categorical fixed factor, in which the 5-punishment condition was the reference group and standard deviations per participant and block was added as a continuous fixed factor. To assess the relationship between the performance in the two different tasks a model was estimated with timing optimality (i.e., the difference between the actual adjustment and the optimal adjustment) as continuous dependent variable, distance optimality as continuous fixed factor, and punishment level as categorical fixed factor.

For each LMM we started with an intercept-only model, including participant as a random factor. We then sequentially added the relevant fixed factors. To test whether a fixed factor improved the model, we calculated Bayes factors using the *lmBF* function from the *BayesFactor* (Version 0.9.12-4.2; Morey & Rouder, 2018) package for R. The default priors of the *BayesFactor* package as described in Rouder and Morey (2012) were used. We will denote the evidence for the alternative hypothesis (H_1_; i.e., the model including the fixed factor) over the null hypothesis (H_0_; i.e., the model excluding the fixed factor) as BF_10_. Fixed factors that yielded a model with a BF over 3 were included in the models (Wagenmakers, 2007).

## Results

### Timing and distance adjustments

**Fig. 3:**
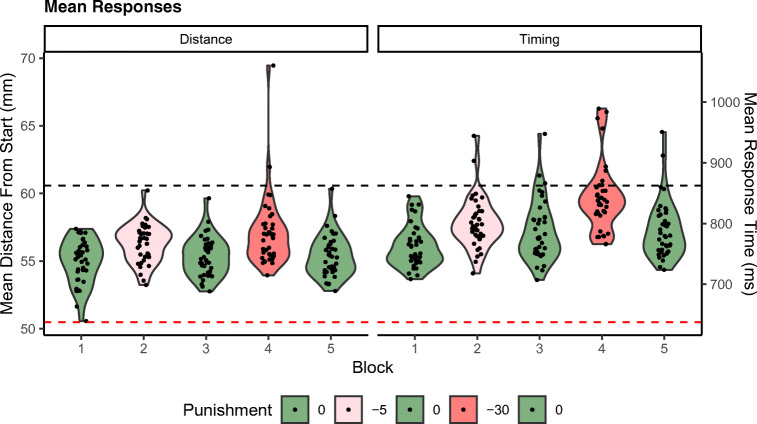
Violin plots representing distributions of mean responses by participant in each of the blocks. The red and black dashed lines represent the lower and upper criterion, respectively; responses below the red line are punished in Block 2 (−5 points) and Block 4 (−30 points). The black dots represent individual mean estimates of participants. (Color figure online)

Figure 3 shows the distribution of the average responses in the different blocks of the timing and distance estimation tasks. The figure shows that participants adjusted their mean responses away from the punished criterion in the punishment blocks (Block 2 and Block 4) in both tasks. Their timing and distance estimations had an efficiency of nearly 100% in the blocks with 0 or 5 points punishment; the median percentage of maximum expected points earned was, respectively, 97% and 95%. In the blocks with 30 points punishment, the median received points equated 82% of the maximum expected amount.

**Fig. 4:**
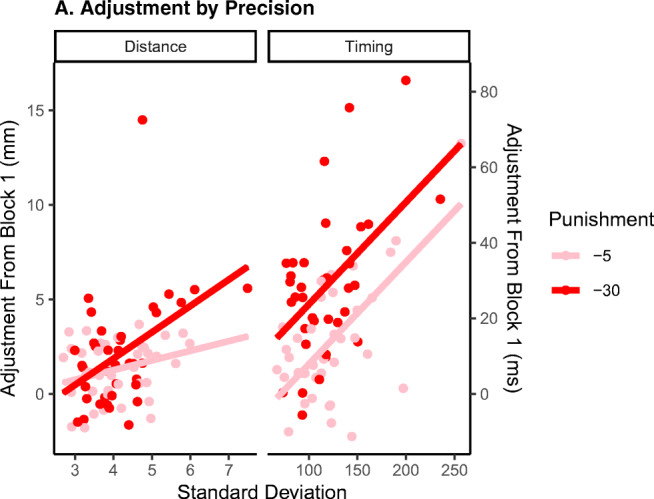
Adjustment in the distance estimation and the timing task in Block 2 and Block 4 relative to Block 1 as a function of a participant’s standard deviation. Imprecise participants adjust their responses relative to the first block more than precise participants did

We hypothesized that imprecise timers and distance estimators would adjust their responses in the blocks with punishment more relative to precise participants. Figure 4 shows that the difference of mean responses between Block 1 and the two blocks with punishment is larger for participants with a high standard deviations. To test this, LMMs predicting the *adjustment of mean responses in blocks with punishment relative to Block 1* were estimated, separately for timing and distance estimation. Adding punishment level and standard deviation as fixed factors improved the model of the timing task significantly (BF_10_ > 1,000 and BF_10_ = 373, respectively), but their interaction did not (BF_10_ = 1.57). The final model coefficients are reported in Table 2. The positive beta coefficients confirm both our primary hypotheses in the timing task: If there was a risk of punishment for being too early, responses were delayed, and noisy timers adjusted their responses more than precise timers did.

**Table 2:** Model coefficients of the two LMMs predicting responses

	Timing				Distance		
	β	*SE*	*p*		β	*SE*	*p*
Intercept	−53.65	19.00	<.01**	Intercept	−0.20	1.81	.62
30-punishment	44.38	6.83	<.01**	30-punishment	−2.92	1.75	.10
*SD*	0.76	0.15	<.01**	*SD*	0.51	0.38	.17
				30-punishment × *SD*	0.88	0.41	.04*

**p < .01, *p < .05

In the distance task, the same modelling procedure was used as for the timing task. The model including only standard deviation as fixed factor was preferred over the intercept only model (BF_10_ = 20), but the data provided no evidence to include punishment level (BF_10_ = 1.38), and moderate evidence to include the interaction between standard deviation and height of punishment (BF_10_ = 2.19). These results are similar to the timing task, although the effect of punishment for responding too close to the center contributed only when it was considered as the interaction with standard deviation (i.e., the effect of *SD* was less pronounced in the low punishment block)

### Within-modality optimality

**Fig. 5:**
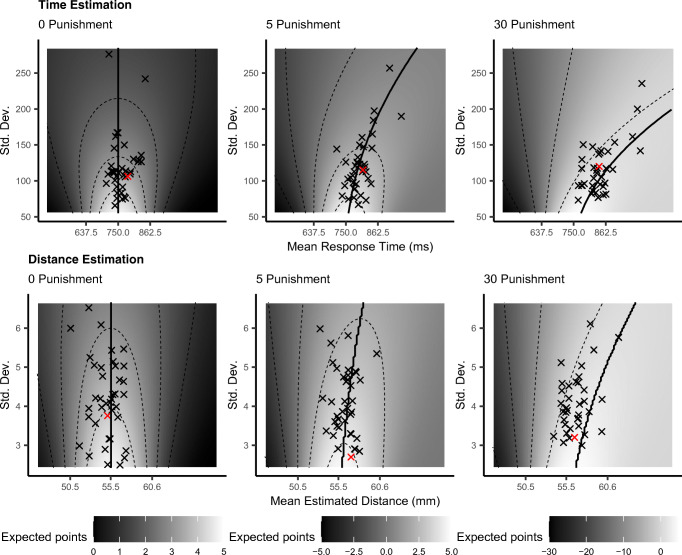
Expected reward surfaces of the timing task (top row) and distance estimation task (bottom row) for Blocks 1 (0-punishment), 2 (5-punishment) and 4 (30-punishment). Mean expected points are calculated with the Expected Reward function and depend on the standard deviation per block, mean response (time or distance), and the height of punishment for being too early or too close. Each “*x*” represents the observed response and the associated standard deviation for an individual participant. The participant marked with a red “*x*” illustrates a response pattern in which adjustment of mean response to the block with 5-punishment was too large, while the adjustment to 30-punishment was too small. The black lines across the ridge of the surfaces indicate optimal mean responses. (Color figure online)

To test whether participants adjusted their responses optimally in the timing and the distance estimation tasks, we calculated optimal responses by maximizing the expected gain function in each block. The “landscapes” of expected gains and the actual responses of participants are shown in Fig. 5, separately for the 0-punishment, 5-punishment, and 30-punishment blocks in both modalities. They demonstrate that, depending on the height of the punishment and the precision of a decision-maker, a ridge of maximum gains emerges. The observed responses can be seen roughly to follow the optimal ridge in the 0-punishment and 5-punishment blocks, but deviate from it in the 30-punishment block. In both the timing task and distance estimation, 81% of the mean responses in the face of 30-points punishment are on the left side of the ridge, indicating these participants did not adjust their mean responses enough to reach their optimal gain, but adopted a risk-seeking strategy. This is also expressed in the efficiency score (i.e., the percentage of points earned in a block relative to the maximum expected amount of points given their *SD*), which in some cases exceeds 100% when the risk-seeking strategy pays off because of “lucky shots.”

**Fig. 6:**
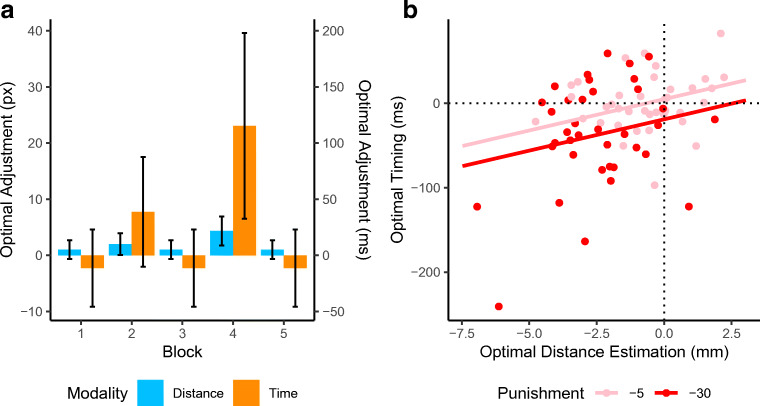
**a** Theoretically optimal adjustments from Block 1 are displayed in the left panel. They illustrate the large variability in the need to adjust main responses (error bars represent one standard deviation). For example, if a participant was already biased towards late/far in Block 1, there is less need to adjust in the face of punishment. **b** Participants generally made optimal adjustments. A positive value on the *y*-axis means that a participant made a too large adjustment of their interval estimation in the blocks with punishment. Positive values on the *y*-axis indicate an overadjustment in the distance estimation in the face of punishment

Figure 6a shows the adjustment participants should make from Block 1 in order to become optimal in another block. The between-participant variability of theoretical optimal adjustments is illustrated by the error bars. For example, it shows that in distance estimation, some participants were already responding so far away from the center in Block 1 (0-punishment), that hardly an adjustment was necessary in Block 2 (5-punishment). Finally, actual mean adjustment from each block compared with Block 1 was subtracted from the optimal adjustments in order to create two variables: timing optimality and distance optimality (see Fig. 6b). This resulted in positive values for blocks in which the participant overadjusted (e.g., was too careful) and negative values for blocks in which the participant should have adjusted more in order to become optimal.

### Cross-modal optimality

Figure 6b shows the relation between a participant’s distance and timing optimality. In order to test whether optimality was correlated across modalities, an intercept-only LMM was estimated with timing optimality as a continuous dependent variable. This model was compared with a model that included distance optimality as a fixed factor. The latter model was highly preferred over the intercept-only model (BF_10_ = 89.00). Adding height of punishment as main effect improved the model (BF_10_ = 3.23), but its interaction with distance optimality did not (BF_10_ = 1.51), showing that the slope of the effect of timing optimality on distance optimality was similar across punishment magnitudes. However, the large negative coefficient (see Table 3) of the categorical variable 30-punishment shows that in this condition adjustments in both timing and distance estimation are suboptimal compared with the 5-punishment condition. Together, these results suggest that the more a participant underadjusts in distance estimation, the more that participant will underadjust timing estimations, regardless of the height of the punishment.

**Table 3:** Model coefficients of the LMM predicting timing optimality

	β	*SE*	*p*
Intercept	4.74	8.10	.56
Distance optimality	7.41	3.53	.04*
30-punishment	−23.84	11.10	.04*

**p < .01, *p < .05

## Discussion

We investigated whether responses are optimally adjusted in the face of different reward/punishment schemes in two different tasks: an interval timing task and distance estimation task. In addition, we tested to which extent the patterns of optimality are similar across modalities. In the two tasks, participants earned money for responding within a fixed temporal or spatial region, but could lose money when responding in an adjacent temporal or spatial region (earlier or closer to the start circle). We found that, when risk increased, imprecise participants adjusted their responses more than precise participants did. As expected, given the scalar property of these estimation, larger mean responses also led to larger standard deviations. When the potential punishment was the same amount as the reward, this adjustment led to near optimal responses in both the timing and distance estimation task, in line with previous timing and motor experiments (Akdoğan & Balcı, 2017; Balci et al., 2011; Freestone, Balcı, Simen, & Church, 2015; Maloney et al., 2007), conforming to the patterns predicted by prospect theory (Kahneman & Tversky, 1979). However, when the potential losses were increased (i.e., six times the amount of the reward), responses in the timing and distance estimation task were respectively earlier and closer to the center than optimal, yielding increased penalties and indicating suboptimal adaptation. The difference between the two punishment levels might be related to what Maloney et al. (2007) call implicit and explicit reasoning about probabilities. They found that if the decision-making is implicit, subjects respond close to optimal, but if part of the reasoning is made explicit, subjects exhibit risk aversion. This suggests another mechanism that may be more domain-general than the one adjusting for endogenous noise.

Our hypothesis that the level of underadjustment or overadjustment is correlated across modalities was confirmed. In both punishment conditions there was a correspondence between the modalities, although the main effect of punishment indicated more underadjustment in the 30-punishment conditions. However, no evidence was found to prefer the model that included interaction over the model without interaction. This may be because only a small adjustment is needed in the balanced reward/punishment condition. The results from the 30-point punishment condition suggest that a similar, central adaptive strategy may underlie the behavioral adjustments in both tasks. That is, following Maloney et al. (2007), the 30-point punishment may be resolved by a more explicit reasoning process, which leads to a decision bias that is similar across modalities. However, whereas Maloney et al. reasoned this bias would reflect a risk-averse tendency, the current study showed a response bias in the opposite direction. We speculate that the under-adjustment in the high punishment condition does not reflect risk-seeking, but rather a failure to accurately reason about how much adjustment is needed to control the risk. Another explanation might be that in risky situations decision-makers rely more on System 1 reasoning, which is more prone to risk-seeking or risk-avoiding biases (Evans, 2008). In the current study, the general proneness to risk-seeking may be attributed to the notion that the probability of being too early is underestimated. Also, since the distance estimation task did not require a speeded response, participants could potentially take more time to deliberate their action compared with the timing task. However, that does not mean that, before the onset of a timing trial, no time can be taken to deliberate about a plan when to response.

Furthermore, to become optimal requires intended responses to be adjusted based on feedback, which introduces three kinds of feedback biases. An intended “response plan” for a point in time will be earlier or later based on the feedback on the previous trial. If a participant is imprecise enough, the optimal response plan could entail that this point is beyond the reward region in the high punishment condition. Consequently, the feedback a participant will “optimally” get is “too late, 0 points” on most trials and an occasional “correct, +5 points.” Secondly, the participant is unable to know *how much* too late the response was, which makes calibration to an optimal response plan more difficult. Third, seeing “too late” on a couple of consecutive trials may incite earlier responses despite the response in fact being optimal. These feedback biases, introduced by experimental settings, raise the question of what to consider true optimal behavior.

It has been suggested that describing the results from perceptual decision-making studies as (sub)-optimal should be avoided (Rahnev & Denison, 2018). A good argument for this statement is that if one observes behavior that is nonnormative, it may simply be because the model of normative—“optimal”—behavior does not describe all the factors of the task well enough. Therefore, Rahnev and Denison (2018) argue for an approach that shifts from *ideal observer models* towards *standard observer models*. A standard observer model not only incorporates optimal decision rules but also combines these with other plausible decision rules. In response to this argument, Simen and Balcı (2018) note that optimality may be at the core of any standard observer model and that this is especially true in timing tasks, in which evidence for optimality is often observed. In that respect, we show that optimally can be found in both domains with small penalties, but not in either domain with larger penalties. Even though based on these results we cannot argue that adjustment is identical, the interaction that would be expected on the basis of Simen and Balcı’s work is not observed.

In conclusion, we demonstrated that participants are able to use near optimal strategies based on their level of precision in both temporal and distance estimation. In addition, we found evidence that these strategies might be modality independent. However, a suboptimal response bias was observed in both tasks when the punishment was significantly higher than the potential reward, which could potentially be ascribed to explicit reasoning processes. Understanding the nature of these biases is instrumental for the development of standard observer models.

### Open practices statement

All data and materials are available (https://osf.io/yb7ew/).
